# Subsocial Cockroaches *Nauphoeta cinerea* Mate Indiscriminately with Kin Despite High Costs of Inbreeding

**DOI:** 10.1371/journal.pone.0162548

**Published:** 2016-09-21

**Authors:** Sofia Bouchebti, Virginie Durier, Cristian Pasquaretta, Colette Rivault, Mathieu Lihoreau

**Affiliations:** 1 Research Center on Animal Cognition (CRCA), Center for Integrative Biology (CBI), University of Toulouse, CNRS, UPS, Toulouse, France; 2 CNRS UMR 6552 Ethologie Animale et Humaine, Université de Rennes 1, Avenue du Général Leclerc, Rennes, France; University of Vienna, AUSTRIA

## Abstract

Many animals have evolved strategies to reduce risks of inbreeding and its deleterious effects on the progeny. In social arthropods, such as the eusocial ants and bees, inbreeding avoidance is typically achieved by the dispersal of breeders from their native colony. However studies in presocial insects suggest that kin discrimination during mate choice may be a more common mechanism in socially simpler species with no reproductive division of labour. Here we examined this possibility in the subsocial cockroach *Nauphoeta cinerea*, a model species for research in sexual selection, where males establish dominance hierarchies to access females and control breeding territories. When given a binary choice between a sibling male and a non-sibling male that had the opportunity to establish a hierarchy prior to the tests, females mated preferentially with the dominant male, irrespective of kinship or body size. Despite the lack of kin discrimination during mate choice, inbred-mated females incurred significant fitness costs, producing 20% less offspring than outbred-mated females. We discuss how the social mating system of this territorial cockroach may naturally limit the probability of siblings to encounter and reproduce, without the need for evolving active inbreeding avoidance mechanisms, such as kin recognition.

## Introduction

Inbreeding–the reproduction of closely related individuals–increases homozygosity and the expression of deleterious recessive alleles, often resulting in a reduction of fitness traits in the progeny known as inbreeding depression [1,2]. On the other hand, theoretical models also predict that inbreeding can have substantial positive effects on the parent’s inclusive fitness by increasing their representation of genes identical by descent in future generations [3–5]. As the costs of inbreeding depression and the kin-selected benefits of inbreeding do not necessarily cancel out, their balance is expected to determine whether animals should actively avoid or favour mating with their kin [3–7].

As such, many animal species that incur particularly high costs of inbreeding depression, from snails to primates, have evolved strategies to avoid inbreeding [8,9]. These include pre-copulatory mechanisms that reduce the probability of mating with kin, such as the asynchronous maturation of the sexes [10,11], the dispersal of individuals from their native group [12–15], the copulation of females with several males (polyandry) [16–18] or kin discrimination during mate choice [19,20]. Alternatively, post-copulatory mechanisms can reduce the fertilisation success of inbred matings, such as male-female gamete incompatibility [21,22] or cryptic female choice (e.g. when females prevent complete ejaculation, discard sperm, or reduce the number of offspring produced) [23–26]. These mechanisms are particularly important in group-living animals, when individuals are the most likely to encounter close relatives and mate with them [2,8,9].

Arthropods are no exception [27]. In the advanced eusocial insects, characterised by overlaps of adults generations, cooperative brood care and reproductive division of labour [28], inbreeding avoidance is typically achieved via the dispersal of reproductive individuals (gynes and/or males) from their native colony (e.g. ants [29–31], honeybees [32], wasps [33], termites [34]; but see [35,36]). However, much less is known about inbreeding avoidance in the vast majority of less social (presocial, sensu [28]) arthropod species that exhibit no reproductive division of labour and only low levels of cooperation [37,38]. Studies in presocial insects (e.g. leaf beetles [39], field crickets [40], domiciliary cockroaches [41]) and some other arthropods (e.g. spider mites [42]) suggest that kin recognition–the ability to discriminate conspecifics based on relatedness [43]–is a common mechanism for inbreeding avoidance in socially simpler species. In these social groups where all individuals can potentially reproduce and show limited dispersal, the risks of incestuous matings are particularly high. For instance, in the German cockroach (*Blattella germanica*), that live in large mixed-family aggregations [44], kin recognition enables males and females to reject siblings as potential mating partners [45], thereby avoiding important fitness costs in the form of reduced offspring production [41,46]. Kin recognition is mediated by the perception of quantitative variations of odour profiles (cuticular hydrocarbons) correlated with relatedness, allowing for the discrimination of fine scale differences in genetic similarity [47,48].

The Blattodea (cockroaches and termites) is a relatively overlooked but fundamentally interesting phylogenetic group for comparative research on the evolution of social and mating systems due to their wide spectrum of social lifestyles, from solitary to eusocial species [38,44,49]. Beyond *B*. *germanica*, most knowledge of sexual selection in cockroaches comes from mate choice studies on the subsocial (sensu [28]) species *Nauphoeta cinerea* [50–56]. In *N*. *cinerea*, males establish dominance hierarchies through agonistic interactions [57] and dominant males advertise their status by secreting a sex pheromone attractive to females [58]. Previous studies show that females preferentially mate with dominant males, because they produce larger quantities of sex pheromone and display a more intense sexual courtship [50,51,59] (but see [60]). Although mate choice by *N*. *cinerea* has been intensively studied in the lab, the social and genetic structures of field populations are poorly documented [61], and potential mechanisms involved in the regulation of inbreeding levels still have to be evidenced. In this ovoviviparous cockroach, males and females have a synchronous development [61], females typically mate once [62] (but may occasionally re-mate between producing clutches [63]), and neither males nor females are known to disperse [61]. Therefore we hypothesised that kin discrimination during mate choice would be an efficient mechanism for inbreeding avoidance in this species.

Here we examined this possibility by testing the influence of kinship on mate choice in *N*. *cinerea*. We conducted female mate choice experiments in which we manipulated the kinship and the hierarchical status of males. We then evaluated the consequences of inbreeding by monitoring the lifetime reproductive success of females in the different types of matings.

## Materials and Methods

### Insect culture

Three commercial colonies of 50 *N*. *cinerea* cockroaches each were obtained (Envie Animale, St Jacques de la Lande, France), mixed and cultured in a single rearing cage (80cm long, 30cm large, 120cm high) in which males and females could freely mate (sex ratio 1:1). Cockroaches were maintained under standard laboratory conditions (25°C, 60% humidity, 12:12 light:dark photoperiod, light on at 10:00 pm) with *ad libitum* access to shelters (cardboard cylinders), food (turkey food pellets) and water. The culture was established 18 months (ca. three generations of adults) before the beginning of the experiments.

**Fig 1:**
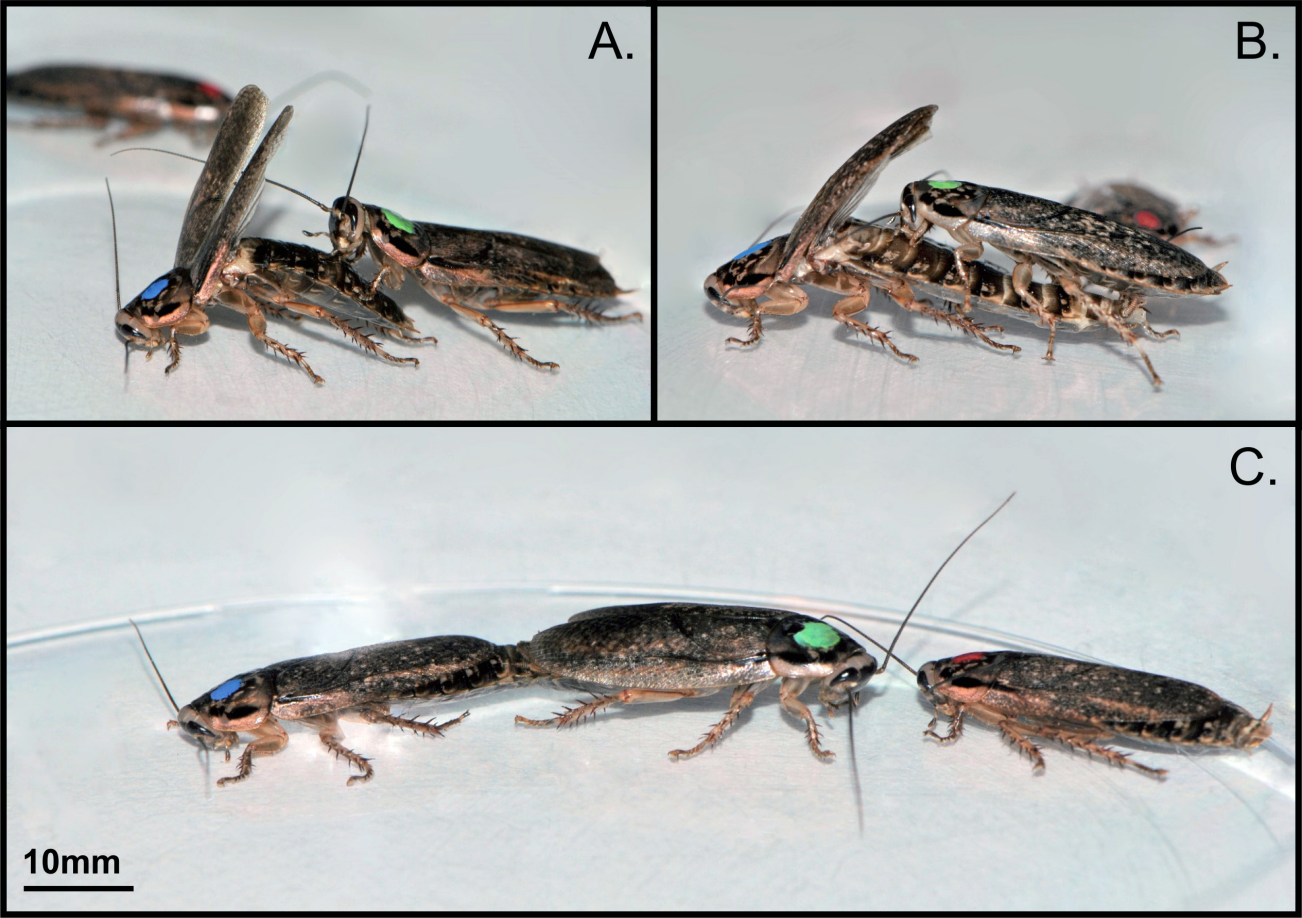
Succession of behavioural acts during mate choice in *Nauphoeta cinerea*. In this example, a virgin female (green dot on the pronotum) is given a choice between two males (blue and red). (A) After having initiated antennal contacts with the female, the courting male (blue) turns around in front of the female and raises its wings so that the female can mount on the male’s abdomen. (B) The female licks the abdominal secretions from the male’s tergal glands while the male pushes his abdomen further back to grasp the female’s genitalia. (C) Copulation lasts for approximately 15 minutes during which the male and the female remain paired in straight-line opposite position, allowing for the formation and transfer of a spermatophore into the female’s genital tract. In this example, the non-copulating male (red) initiates antennal contacts with the female during mating. Photographs by SB.

To obtain experimental subjects of known age and kinship, we collected a random sample of ca. 100 females from the main culture consisting of ca. 100000 individuals and isolated them in individual plastic rearing boxes (8cm Ø, 5cm high) until they produced their first clutch. Nymphs from the same clutch were full-siblings (coefficient of relatedness *=* 0.5) [64]. Nymphs from different clutches were non-siblings of unknown relatedness. Newborn nymphs were transferred into a new rearing box and bred in groups of siblings until adulthood (ca. 100 days). Therefore, in this design, siblings were also familiar and non-siblings were non-familiar. Young adults (one to two days after the imaginal moult) were sexed, uniquely identified with enamel paint on the pronotum (Fig 1), and isolated in individual rearing boxes. Cockroaches were maintained in these conditions for 10 days so that they could reach sexual maturation, while making sure that they all remained virgin until being tested.

### Mate choice experiments

All observations were made under red light (which is not detected by cockroaches [65]) during the cockroach activity peak [66], i.e. the first two hours of the dark phase of the photoperiod (10:00–12:00 am). Tests were conducted in two phases.

During the first phase of the tests, two non-sibling males were introduced into a clear plastic arena (30 cm long, 30 cm large, 8 cm high) and observed for 20 min (*N* = 131 dyads). All interactions between the two males were recorded to calculate a dominance index *D* for each dyad [57]:

$$D=\frac{Im_{1}-Im_{2}}{Im_{1}+Im_{2}}$$

Where *I*_*mi*_ is the total number of agonistic interactions (bites, jumps over) initiated by male *i*. Male 1 was dominant when *D* ≥ 0.75. Male 2 was dominant when *D* ≤ – 0.75. No hierarchy was established when– 0.75 < *D* < 0.75. In this case, the males had no hierarchical status.

In the second phase of the tests, a female (sibling to one of the two males) was introduced in each arena, yielding three types of triads: (1) a female with a dominant sibling and a subordinate non-sibling (*N* = 48 triads), (2) a female with a no-status sibling and a no-status non-sibling (*N* = 34 triads), (3) a female with a subordinate sibling and a dominant non-sibling (*N* = 49 triads). Each triad was observed for 20 min for mating to occur. The 131 females mated within this period. Triads for which mating was initiated but not completed within the 20 min (i.e. the female was still paired to the male) were observed until the end of copulation (range of copulation duration: 6.7–18.5 min, *N* = 131 copulations). For each triad, we recorded the sexual displays of males (antennal contacts with the female, wing raising; Fig 1A), the mating attempts of females (female climbing onto the male’s abdomen and licking abdominal secretions; Fig 1B), the duration of copulation (time spent paired; Fig 1C) and any interference of the non-copulating male with the copulating pair that could have induced a premature end of the copulation [46] (Fig 1C). We never observed any aggressive interactions (bites, jumps over) between males and females before or during mating. During the tests, the experimenter was blind as to the dominance status of males and their relatedness to females.

Trials were stopped immediately after copulation, which guaranteed that each female mated only once. Males were removed from the arena and measured under light CO_2_ anaesthesia. The maximal head width and the length of the left mesothoracic femur were used to estimate body size [67]. Precise measures (± 0.01 mm) were obtained from images of heads and legs taken under a binocular microscope (x 25), using a homemade software [68]. Each cockroach was tested only once.

### Reproductive success of pairs

We observed 131 copulations across six types of pairs (see details in Table 1). Mated females were maintained isolated in the test arenas and moved to a breeding room with *ad libitum* access to food and water. The room was free of males to prevent male pheromones from interfering with the development of clutches [55]. To estimate the reproductive success of pairs, we recorded the number of offspring produced by each female on a daily basis until all females died (survival range: 54–523 days, *N* = 131 females). Newborn nymphs were removed from the arenas to avoid potential differences in the metabolic rate of gestating females due to group size effects (e.g. increased ambient temperature inducing faster gestation in arenas containing large clutches) [68]. During data collection, the experimenter was blind as to whether females had mated with a sibling or a non-sibling male.

**Table 1:** Total number of matings. We observed 131 females mating with six types of males: a dominant sibling, a dominant non-sibling, a no-status sibling, a no-status non-sibling, a subordinate sibling or a subordinate non-sibling. Overall, females mated more often with dominant males than with subordinate males irrespective of kinship.

	Sibling	Non-sibling	Total
Dominant	35 (26.7%)	38 (29%)	73 (55.7%)
No-status	17 (13%)	17 (13%)	34 (26%)
Subordinate	11 (8.4%)	13 (9.9%)	24 (18.3%)
Total	63 (48.1%)	68 (51.9%)	131 (100%)

### Data analyses

All statistical analyses were conducted in R version 2.12.1 [69]. Means are given with standard errors (mean ± SE). Raw data are available in S1 Dataset.

For the dominance analyses, we compared the relative difference in body size (head width, femur length) between the two males of a dyad, in dyads that established a hierarchy and dyads that did not establish a hierarchy, using Wilcoxon tests. For dyads with a hierarchy, we compared the body size of dominant and subordinate males using paired t-tests. We also compared the proportions of dyads in which the dominant and the subordinate males displayed the first agonistic behaviour using an exact binomial test (with a probability of success 0.5). We examined the correlation between the head width and the femur length of males with a Pearson’s product-moment correlation.

For the mate choice analyses, we tested the effect of male dominance status (dominant, subordinate, no-status), male-female kinship (sibling, non-sibling) and male body size (head width) on their frequency of sexual displays (log-transformed to normality) using a generalised linear mixed effect model (GLMM) with triad identity nested within male type as random factor, with the function lmer () in the R package ‘*lme4*’ [70]. We compared all possible models (all combinations of additive and interactive effects) using the Akaike information criterion corrected for small sample size (AICc) by applying the Maximum likelihood (ML) method and selected the model with the lowest AICc [71]. We tested the effect of male dominance status, male-female kinship and male body size on female mate choice (mated, non-mated) using a GLMM with triad identity as random factor, using the function glmer () in ‘*lme4*’. Binomial models were checked for overdispersion and considered valid when the residual deviance was within ±10% of the residual degree of freedom. We ran another multi-model inference based on AICc to assess the best fitted model for our data following the procedure previously described. To take into account the nested structure of the data, we applied pairwise comparison tests to analyse the differences occurring among all the classes of our categorical predictors to both models of frequency of sexual displays and of female mate choice with Tukey’s honest significant difference (HSD) tests in the *‘multcomp’* package in R [72]

We used exact binomial tests (with a probability of success 0.5) to compare the proportion of matings with dominant and subordinate males. We ran three-ways ANOVAs to test the influence of male hierarchical status, male body size or male-female kinship on the latency to copulation (log-transformed to normality) and on copulation durations. We compared the effects of male dominance status and male-female kinship on the frequency of interference behaviour using a generalized linear model (GLM) with binomial errors and an ANOVA on the selected model (using the Wald Chi-Square statistics) after controlling for overdispersion (see above). We compared the duration of copulations with and without interference using a t-test.

For the reproductive success analyses, we compared female longevity across the six types of matings using a Cox regression analysis with the function coxph() in the package ‘*survival*’ [73]. We compared the proportion of fertile and sterile matings in inbred and outbred mating using a Pearson’s Chi-Square test, and the copulation duration between fertile and sterile matings using a Wilcoxon test. We used a four-ways ANOVA to test the effect of female survival, male-female kinship, copulation duration, and male hierarchical status on the total number of clutches produced by females. We made pairwise comparisons of copulation durations, female survival, total number of clutches produced and total number of nymphs produced using t-tests. For multiple comparisons of the number of nymphs produced in each clutch, we used a Bonferroni correction.

## Results

### Dominance hierarchies

Two males were placed in a test arena and their interactions were recorded for 20 min (*N* = 131 dyads). By the end of the observations, dominance hierarchies were established in 74% of the dyads (*N* = 97/131; Table 1). In the other 26% of the dyads (*N* = 34/131), males were considered to have no hierarchical status.

On average, the two males of a dyad had a body size that differed by 4% (head width difference: 4.6 ± 0.3%; femur length difference: 5.2 ± 0.4%; *N* = 131 dyads). This relative difference in body size was similar whether males established a hierarchy or not, both for head width (hierarchy: 4.7 ± 0.4%, *N* = 97 dyads; no hierarchy: 4.4 ± 0.6%, *N* = 34 dyads; Wilcoxon test, W = 1741, P = 0.631) and femur length (hierarchy: 5.2 ± 0.5%, *N* = 97 dyads; no hierarchy: 5.3 ± 0.6%, *N* = 34 dyads; Wilcoxon test, W = 1529, P = 0.53). When only considering the 97 dyads in which a hierarchy was established, we found no difference in head width (dominant: 4.33 ± 0.02 mm; subordinate: 4.31 ± 0.02 mm, *N* = 97 dyads; paired t-test, t_96_ = -0.56, P = 0.578) nor femur length (dominant: 4.65 ± 0.02 mm, subordinate: 4.63 ± 0.02 mm, *N* = 97 dyads; paired t-test, t_96_ = 0.45, P = 0.654) between dominant and subordinate males.

Analysis of the sequences of male-male interactions indicates that the male that displayed the first agonistic behaviour became dominant in 83.5% of the dyads (dominants: *N* = 81 dyads, subordinates: *N* = 16 dyads, binomial test, P < 0.001). These results confirm previous observations that the dominance status of *N*. *cinerea* males is not determined by body size [74], but merely by their tendency to initiate the fight [75]. Since our measures of head width and femur length were highly correlated (Pearson’s product-moment correlation, R = 0.81, t_260_ = 22.01, P < 0.001), we only used head width as a proxy of body size in the following analyses.

### Mate choice

A female was introduced in each arena containing two males and triads were observed for another 20 min. The total numbers of matings with each type of male are summarised in Table 1. On average, dominant males expressed more sexual displays (1.78 ± 0.16, *N* = 97 males) than no-status males (1.09 ± 0.16, *N* = 68 males) and subordinate males (0.64 ± 0.16, *N* = 97 males). This result held true when weighting for the nested structure of the data (Tukey HSD: dominants vs. no-status: estimate = 0.538, SE = 0.193, P = 0.015; dominants vs. subordinates: estimate = 1.193, SE = 0.201, P <0.001; no-status vs. subordinates: estimate = 0.655, SE = 0.225, P = 0.010). Neither male-female kinship nor male body size had an effect on male sexual displays (Table 2). Female mate choice was significantly influenced by male dominance status but not by male-female kinship nor male body size (Table 2; Tukey HSD: dominant vs. no-status: estimate = 1.113, SE = 0.338, P = 0.002; dominant vs. subordinates: estimate = 2.227, SE = 0.333, P <0.001; no-status vs. subordinates: estimate = 1.113, SE = 0.338, P = 0.002). Accordingly, when considering only the 97 triads in which males established a dominance hierarchy, females mated significantly more often with the dominant male than with the subordinate male (dominants: *N* = 73 matings, subordinates: *N* = 24 matings, binomial test, P < 0.001).

**Table 2:** Multi-model selection procedure. We compared generalised linear mixed effects models (GLMM) with triad_ID nested in male type as random effect and the following combination of fixed effects: male dominance status (Dominance), kinship (Kin), male body size (Body Size), interaction between male dominance status and kinship, interaction between male body size and kinship, interaction between male body size and male dominant status. Number of parameters retained (K), Akaike information criterion weighted for small sample size (AICc), differences between AICc (Δ AICc) and normalized Akaike weights (wi) for each model are shown. Data are showed for sexual display and mate choice analyses.

**Multi-model selection procedure for SEXUAL DISPLAY**	K	AICc	Δ AICc	wi
Dominance	5	753.8	0	0.467
Dominance + Kin	6	755.7	1.92	0.179
Dominance + Body Size	6	755.7	1.98	0.173
Full model	12	767.0	13.26	0.001
Null model	3	769.4	15.63	0
**Best model (Dominance)**	Estimate	SE	P	
Intercept	0.339	0.119	0.004	
Dominance (N)	-0.537	0.193	0.005	
Dominance (S)	-1.193	0.201	<0.001	
**Multi-model selection procedure for MATE CHOICE**	K	AICc	Δ AICc	wi
Dominance	4	319.5	0	0.383
Dominance + Body Size	5	320.9	1.42	0.188
Dominance + Kin	5	321.2	1.70	0.164
Full model	11	330.8	11.31	0.001
Null model	2	367.3	47.75	0
**Best model (Dominance)**	Estimate	SE	P	
Intercept	1.112	0.235	<0.001	
Dominance (N)	-1.112	0.338	<0.001	
Dominance (S)	-2.225	0.332	<0.001	

**Fig 2:**
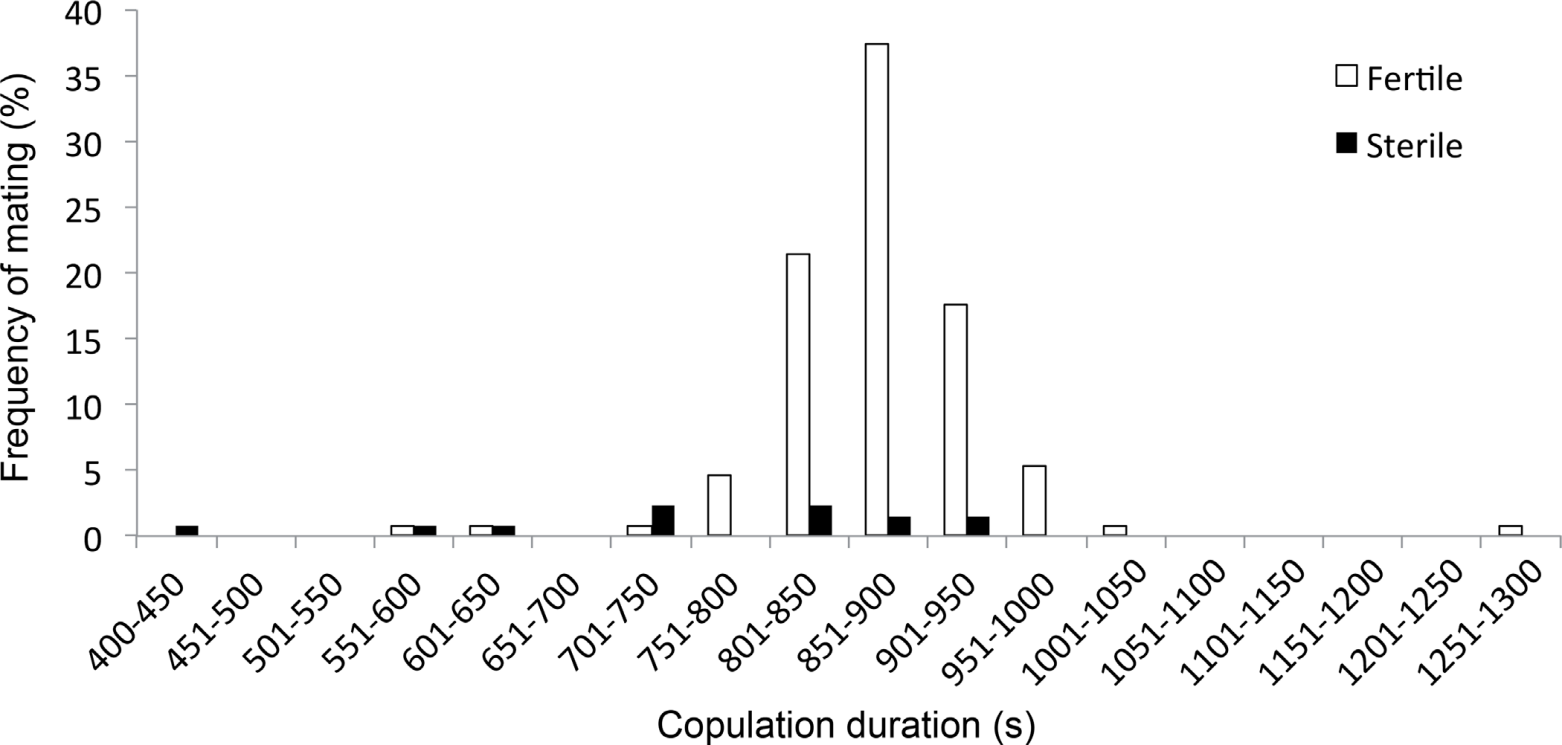
Frequency of fertile matings (white bars) and sterile matings (black bars) in relation to copulation duration. Females that copulated < 550 s never produced nymphs. Only 33% (*N* = 3/9) of the copulations that lasted ≤ 750 s were fertile in contrast to 94% (*N* = 115/122) for copulations that lasted > 750 s. *N* = 131 matings.

The dynamics of mate choice were similar across the six types of triads. On average, copulation started 227 ± 23 s (*N* = 131 matings) after the introduction of the female in the arena. The latency to copulation was neither influenced by male hierarchical status, nor by male body size, or by male-female kinship (Three-ways ANOVA, dominance status: F_2,123_ = 0.17, P = 0.847; head width: F_1,123_ = 0.01, P = 0.963; kinship: F_2,123_ = 0.92, P = 0.401; dominance status x head width: F_2,123_ = 2.4, P = 0.095). Although time spent copulating varied considerably between triads (range: 400 s– 1278 s; Fig 2), we found no effect of male dominance status, male body size or male-female kinship on copulation duration (Three-ways ANOVA, dominance status: F_2,123_ = 0.78, P = 0.46; head width: F_1,123_ = 0.18, P = 0.674; kinship: F_2,123_ = 0.1, P = 0.372; head width x kinship: F_2,123_ = 3.07, P = 0.06).

The second (non-copulating) male interfered with the copulating pair in 42% of the triads (*N* = 55/131), by directing agonistic interactions (bites, jumps over) towards the pair. This behaviour was more frequently displayed by dominant males (70.8% of trials when females mated subordinates, *N* = 17/24), than by no-status males (47.1% of trials with triads with no hierarchy, *N* = 16/34) or by subordinate males (30.1% of trials when females mated with dominants, *N* = 22/73), irrespective of their kinship with the female (GLM with Binomial errors, dominance status: Deviance = 165.22, df = 2,126, P = 0.002; kinship: Deviance = 178.10, df = 2,128, P = 0.939; dominance status x kinship: Deviance = 161.19, df = 3,123, P = 0.258). Copulations with interferences lasted significantly less than copulations without interferences (with interference: 825 ± 12 s, *N* = 55 triads; without interference: 887 ± 9 s, *N* = 76 triads; t-test: t_90,3_ = -3.68, P < 0.001). Presumably in these triads the presence of the second male disturbed and prematurely interrupted mating.

### Reproductive success

Immediately after mating, males were removed from the arenas and females were maintained in isolation until they died (325 ± 8 days, *N* = 131 females) to record their lifetime offspring production. The survival of females was neither affected by male dominance status, nor by male body size, male-female kinship, or copulation duration (Analysis of Deviance on a Cox model; dominance status: χ^**2**^ = 0.18, df = 2, P = 0.91; head width: χ^**2**^ = 2.77, df = 1, P = 0.096; kinship: χ^**2**^ = 0.27, df = 2, P = 0.529; copulation duration: χ^**2**^ = 0.93, df = 1, P = 0.335).

Ninety per cent of the matings (*N* = 118/131) were fertile (i.e. producing at least one nymph). This proportion was similar for inbred matings and outbred matings (inbred: *N* = 9/63, outbred: *N* = 4/68, Pearson’s Chi-Square test: χ^**2**^ = 1.73, df = 1, P = 0.189). However, copulation duration was significantly longer for fertile matings than for sterile matings (fertile: 875 ± 7 s, *N* = 118 matings; sterile: 735 ± 46 s, *N* = 13 matings; Wilcoxon test: W = 345.5, P = 0.001). Only 33% (*N* = 3/9) of the copulations that lasted 750 s or less were fertile compared to 94% (*N* = 115/122) of the copulations that lasted longer than 750 s (Fig 2).

**Fig 3:**
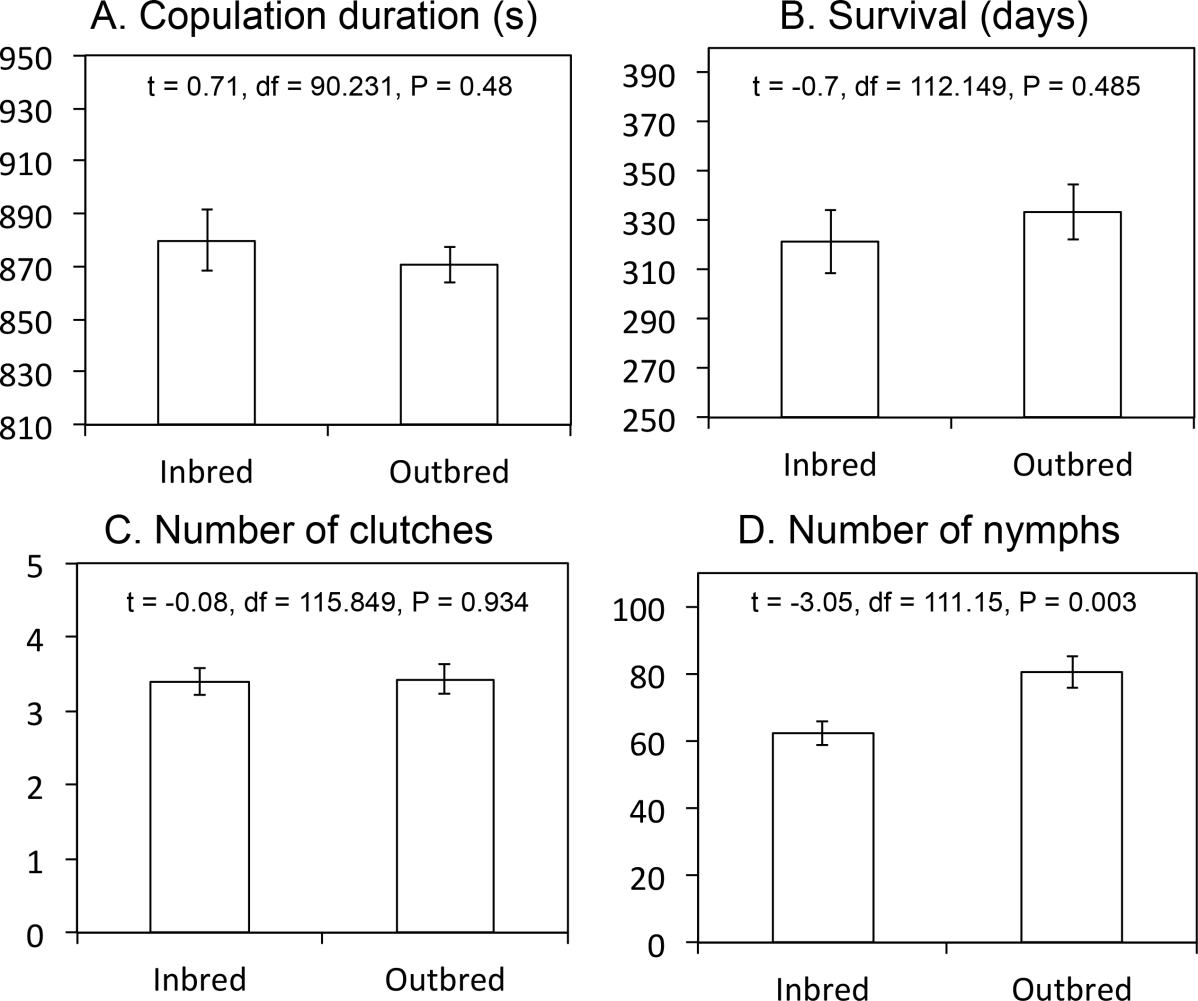
Reproductive success of pairs in inbred and outbred matings. Kinship between males and females neither affected (A) copulation duration, (B) female survival, nor (C) the total number of clutches produced. (D) Inbred-mated females produced a total of 20% less offspring than outbred-mated females during their entire lifetime. Means are showed with standard errors (mean ± SE). *N* inbred = 56 females. *N* outbred = 61 females.

**Fig 4:**
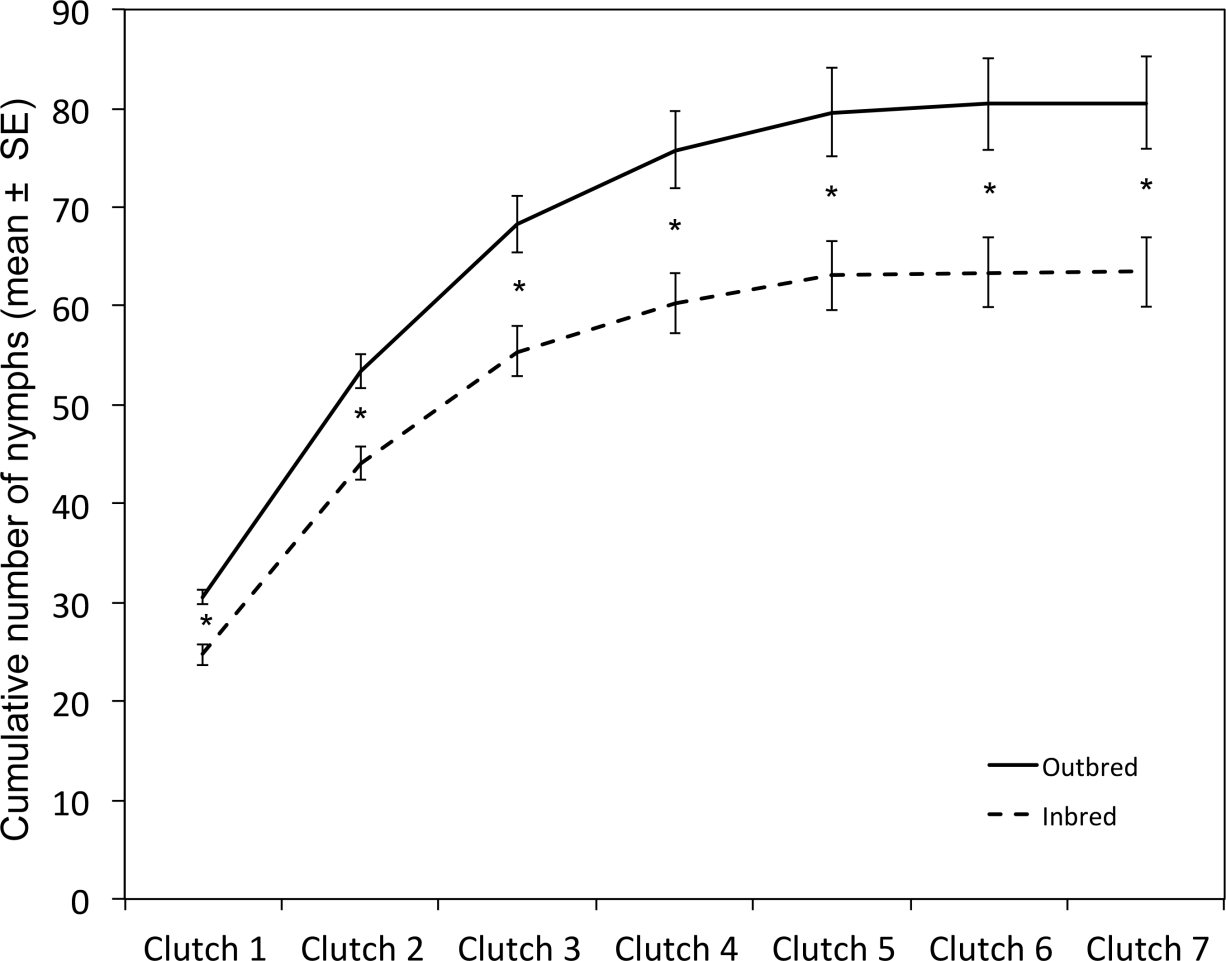
Dynamics of nymph production for inbred and outbred matings. Inbred-mated females produced 20% less nymphs than did outbred-mated females. This difference could be observed from the first clutch on and gradually increased as females produced more clutches. *N* inbred = 56 females. *N* outbred = 61 females. * P < 0.007 (pairwise comparisons with t-tests after a Bonferroni correction; clutch 1: t_94.362_ = -4.52, P < 0.001; clutch 2: t_114.664_ = -3.83, P < 0.001; clutch 3: t_113.976_ = -3.38, P < 0.001; clutch 4: t_110.14_ = -3.17, P = 0.002; clutch 5: t_110.095_ = -2.92, P = 0.004; clutch 6: t_109.288_ = -2.91, P = 0.004; clutch 7: t_109.244_ = -2.90, P = 0.004).

On average, females of the 118 fertile matings produced 3.42 ± 0.14 clutches. This total number of clutches produced by females was positively influenced by survival, but not by male-female kinship, copulation duration, or male dominance status (Four-ways ANOVA, survival: F_1,110_ = 66.8, P < 0.001; kinship: F_2,110_ = 1.3, P = 0.277; copulation duration: F_1,110_ = 0.03, P = 0.856; dominance status: F_2,110_ = 2.95, P = 0.057). The total number of nymphs produced by females was not only affected by their survival but also by their kinship with males (Four-ways ANOVA, survival: F_1,108_ = 15.51, P < 0.001; kinship: F_2,108_ = 5.25, P = 0.007; copulation duration: F_1,108_ = 0.06, P = 0.814; dominance status: F_2,108_ = 2.35, P = 0.1; survival x dominance status: F_2,108_ = 3.06, P = 0.051). Therefore, although inbred-mated and outbred-mated females had similar copulation durations (t-test, t_90.231_ = 0.71, P = 0.48; Fig 3A), similar survival (t-test: t_112.149_ = - 0.7, P = 0.485; Fig 3B), and produced similar total numbers of clutches (t-test, t_115.849_ = - 0.08, P = 0.934; Fig 3C), mating with a sibling reduced females’ lifetime nymph production by 20% (t-test: t_111.15_ = -3.05, P = 0.003; Fig 3D). This negative effect of inbreeding on nymph production was significant from the first clutch (t-test, t_94.362_ = -4.52; P < 0.001) and accumulated with the production of all subsequent clutches (Fig 4).

## Discussion

We examined the influence of kinship on mate choice in the subsocial cockroach *N*. *cinerea*. Using binary choice tests, we found that females mated preferentially with dominant males irrespective of their relatedness to them and despite significant costs of inbreeding on offspring production. Presumably, the social mating system of this territorial cockroach species limits the probability of sexually receptive siblings to encounter and mate, without the need for evolving kin recognition.

*N*. *cinerea* is a model species for research on insect sexual selection. Numerous previous studies showed that the competitive interactions between *N*. *cinerea* males [50–53,59], their production of sex pheromones [60,76] and their mating history [56,77] are key factors for female mate choice. Our analyses of male-male interactions corroborate these findings, showing that the dominance status of males is not determined by differences in body sizes [74] but mainly by their propensity to initiate the fight with competitors [75]. The resulting mate choices also confirm that dominant males express more sexual displays [74], and obtain significantly more matings than their subordinates, thereby emphasizing the fundamental role of male hierarchies in sexual selection for this species [50,51,59]. Building on these results, our study demonstrates that female mate choice is independent of their kinship with males. Thus contrary to *B*. *germanica*, where both sexes perform a mutual mate choice to avoid inbreeding [41,45,46], *N*. *cinerea* adults do not exhibit any detectable form of pre-copulatory kin discrimination, be it at the level of partner choice, female acceptation latency of males, or copulation duration. Yet, mating with a sibling reduced the reproductive success of pairs, as illustrated by the 20% decrease of the total numbers of nymphs produced by inbred-mated females. This effect of inbreeding, observable from the first generation of offspring, is comparable the ca. 12% decrease measured in *B*. *germanica* using a similar experimental approach [41,45,46]. Although our measure of reproductive success cannot rule out whether these observed inbreeding effects are caused by a reduced gametic compatibility (less embryos produced), an increased developmental failure of the embryos (less viable nymphs produced) or both, inbreeding depression in *B*. *germanica* induces higher rates of embryo abortion [46], thus primarily supporting the hypothesis of a developmental failure.

The duration of copulation also had important consequences on offspring production in both inbred and outbred matings (33% of fertile matings for copulations ≤ 750 s in our experiment) suggesting that the production of a spermatophore (capsule containing sperm) by the male and its transmission to the female require a minimum mating duration. This is consistent with previous observations by Roth [62] reporting that the spermatophores of *N*. *cinerea* males were fully transferred and firmly inserted in the female’s genital tract in only 27% of the copulations experimentally interrupted between 600 and 720 s. Interestingly, the non-copulating males frequently engaged in agonistic interactions towards the copulating pair, which tended to disturb copulations, reduce their duration, and thus potentially impact on the reproductive success of pairs. These competitive interactions during mating could explain why the durations of copulations by dominant males were not longer than those by subordinate males, a difference with previous mate choice studies that involved only one male [53] or when a second (non-copulating) male was physically separated from the copulating pair [50]. Again we found no evidence of kin discrimination during these interactions as non-copulating males interfered indiscriminately with all pairs, even though they potentially imposed a cost to their sisters by interrupting their copulation and thus reducing the probability of mating success. Whether such competitive interactions during mating are frequent in populations of freely interacting cockroaches, where more than two males may compete for the same female and unsuccessful males are free to stay or escape territories remains to be confirmed.

So why do these cockroaches mate indiscriminately? The relatively high costs of inbreeding depression observed in our study suggest that other mechanisms than kin recognition could mediate inbreeding avoidance in *N*. *cinerea*. Post-copulatory mechanisms such as cryptic female choice [78] are unlikely because females do not mate with multiple males in the same reproductive cycle [54,62]. Nonetheless, it remains to be tested whether inbred-mated females can use potential re-mating opportunities between producing clutches [63] to mate with non-kin and mitigate the effects of inbreeding on their future clutches. Levels of mate choosiness may vary with age and future mating opportunities [79,80], as for instance in the field cricket *Gryllus lineaticeps* where younger females tend to be less choosy than older females [81].

Another possibility is that the structure of the *N*. *cinerea*’s social mating system itself provides pre-copulatory mechanisms that naturally limits encounters between sexually receptive kin and reduces the risks of inbreeding. For instance, the tendency of males to compete and establish dominance hierarchies ([66,82] see also this study) may induce some level of male dispersal from their native group. Although in artificially crowded confined space, such as laboratory or commercial cultures that can contain thousands of cockroaches per cubic meter, males show intense dominance interactions (reaching almost linear hierarchies where high rates of agonistic behaviour and numerous turn-overs of top-ranking males occur) [57,83], population densities are likely much lower in field conditions. Under low population densities (ca. 100 males/m^3^), males tend to occupy all the available space to establish non-overlapping territories, thus reducing the probability of sexually mature siblings to encounter [82]. Additionally, while females reproduce between their third and their sixth day post adult moult [62,84], only older males, aged over six days post adult moult, can potentially become dominants and therefore competitive for accessing mating partners [74]. The apparent mismatch between the reproductive dynamics of males and females may reduce the frequency of matings between siblings from the same clutch. It is also unlikely that males mate with siblings from different clutches since they remain dominants (and thus competitive for mating) for only two to three weeks [82] while the gestation time necessary for a female to produce two successive clutches typically lasts longer (e.g. 40 days at 28°C [77]).

Alternatively, it is possible that the costs of inbreeding are counterbalanced by other benefits until yet rarely considered in this species. Many animals tolerate incestuous mating despite severe inbreeding depression (great tits [85,86], dolphins [87], yellow-bellied marmots [88], house sparrows [89]), for instance when the probability to find unrelated mates is low [89,90] or when mating with siblings provides kin-selected benefits in the form of increased genetic representation in future generations [3–7]. In *N*. *cinerea*, females benefit from mating with dominant males that tend to produce more dominant sons [53]. Therefore it could be more advantageous for a female to mate with a dominant kin (that will provide less offspring but more dominant sons) rather to mate with a subordinate non-kin (that will provide more offspring but less dominant sons). Further experiments detailing the typical size, genetic composition and spatial structure of *N*. *cinerea* populations are needed to clarify the social ecology of these cockroaches and test these alternative hypotheses. Comparing the behaviour of cockroaches from different populations, in the lab and in the field, will be critical to address these questions as illustrated, for instance, by the contrasting results on kin recognition and inbreeding avoidance reported in various *Drosophila* strains (e.g. [91–95]).

Evidence for kin recognition in presocial insects is scarce (e.g. [39,41]) but has also received relatively little attention [20,27,37,38]. Although our experiments could not demonstrate a discrimination between siblings and non-siblings during mate choice, it does not definitely rule out the existence of kin recognition in *N*. *cinerea*. For instance, kin discrimination may be observed in a different context when the costs of discrimination errors could be higher than the observed inbreeding depression, as for instance for avoiding kin cannibalism during periods of starvation [43,96,97]. Context dependent kin discrimination is common in the animal kingdom and has been modelled in the form of an acceptance threshold response [43,96,97]. For example, in the eusocial colonies of paper wasps *Polistes dominulus* [98] and Argentine ants *Linepithema humile* [99], aggression of non-kin (i.e. non-nestmates) is only observed in the presence of cues indicating the proximity of the colony (e.g. nestmates, nest fragments), a context when the costs of discrimination errors are the highest due to the risk of accepting intruders in the nest. Context dependent discrimination can also be expressed at different developmental stages as in the subsocial European earwig *Forficula auricularia* where kin recognition odours are masked during the early developmental stages to prevent nepotistic conflicts between juveniles of different patrilines, but are revealed in adults to avoid incestuous mating [100]. Although relatively few studies have investigated social recognition systems in the Blattodea, more or less accurate forms of kin recognition have been described in subsocial [101], communal [41,45,46] and eusocial [102] species. Further examination of these mechanisms in cockroach and termite species presenting various levels of social complexity hold considerable promises for exploring the evolutionary relationships between communication, mating systems and social structures in arthropod societies.

## Supporting Information

S1 Dataset Raw data. (XLSX)
